# The Importance of IgG Avidity and the Polymerase Chain Reaction in Treating Toxoplasmosis during Pregnancy: Current Knowledge

**DOI:** 10.1155/2013/370769

**Published:** 2013-09-26

**Authors:** João Bortoletti Filho, Edward Araujo Júnior, Natália da Silva Carvalho, Talita Micheletti Helfer, Priscila de Oliveira Nogueira Serni, Luciano Marcondes Machado Nardozza, Antonio Fernandes Moron

**Affiliations:** Department of Obstetrics, Federal University of São Paulo (UNIFESP), Rua Carlos Weber, 956 Apartment, 113 Visage, Vila Leopoldina, 05303-000 São Paulo, SP, Brazil

## Abstract

A brief report on the nature and epidemiology of *T. gondii* infection is firstly presented. The importance of the specific IgG avidity test and polymerase chain reaction (PCR) for toxoplasmosis is discussed, along with their significance and importance as auxiliary methods for determining the most likely time for the initial infection by this coccidian and for defining the therapeutic strategy. Lastly, practical comments are made in relation to the classical therapeutic regimens, with special attention to the indications for fetal treatment, when this is necessary.

## 1. Introduction

Toxoplasmosis is an infection caused by the intracellular protozoon *T. gondii*, for which the primary host is cats. It is known that approximately 10% of all cats are contaminated by *T. gondii*. Sexual reproduction of this parasite takes place in the intestinal environment of cats, and the oocysts produced are eliminated together with the feces. The oocysts remain viable in the external environment for up to one year.

Within this setting, *T. gondii* contaminates secondary hosts through these hosts' contact with sand, foods, or plants that have been contaminated by the oocysts. After ingestion of the oocysts, the oocyst wall becomes thinned by the digestive juices, thereby leading to release of sporozoites into the intestinal lumen. The sporozoites rapidly invade the intestinal epithelium, though the vascular endothelium. At this stage of the evolution, they are transformed into tachyzoites, which are the invasive form of the parasite. Through tachyzoite dissemination, the parasite will lodge in a wide variety of tissues in the secondary host, in which large numbers of cysts that are rich in bradyzoites are produced. The secondary hosts are commonly humans, rodents, birds, crustaceans, domestic animals, and practically all other warm-blooded animals, which explains why consumption of raw or undercooked meat can contaminate other hosts with bradyzoites from tissue cysts. Ingestion of sporozoites occurs mainly through eating poorly washed fruits or vegetables, doing gardening, coming into contact with children who were playing on the ground or on sand, and so on [1].

Data in the literature consistently draw attention to the importance of proper guidance regarding the commonest means of becoming infected with *T. gondii*, with the aim of preventing this event within the pregnancy cycle and the possible harm done to the fetus [2, 3]. A study conducted in the “Economic Research Service” in the United States concluded that approximately half of the cases of toxoplasmosis occurred due to consumption of raw or undercooked meat and that the annual cost of treating such cases in that country was approximately U$ 7.7 billion. This high amount was due mainly to the congenital complication resulting from vertical transmission of the parasite to the fetus [4].

Although toxoplasmosis is extremely benign among immunocompetent women, there is a risk of vertical transmission of the parasite when this infection occurs during pregnancy. The risk increases with advancing gestational age in the following manner: first trimester 10–25%, second trimester 30–50%, and third trimester 60–90%. This seems to occur as a function of the increase in the mass of the placenta, thus increasing the tissue available for tachyzoites to invade. On the other hand, the increase in the volume of the placenta also contributes towards increasing the quantity of cysts rich in bradyzoites, which implies a greater risk of fetal infection in cases of reactivation of the pathological condition. However, the congenital complications become greater with earlier infection during pregnancy [5].

## 2. Serological Diagnosis

Investigation of IgG and IgM levels should be incorporated into the prenatal routine for the commonest infections and for teratogenic infections, including rubella, cytomegalovirus, and toxoplasmosis. 

Diagnoses of acute or progressive toxoplasmosis infection are based on detection of its specific antibodies, with the aim of making an early diagnosis of maternal seroconversion and instituting appropriate treatment. This is extremely important for preventing vertical transmission of *T. gondii *and its deleterious effects on the fetus [6–9]. Findings of toxoplasmosis-specific IgM indicate occurrences of acute infection, although these antibodies may be present in the maternal serum from one week to 18 months after the date of invasion by the parasitic tachyzoites. Specific IgG appears in the maternal serum between one and two months after the initial infection.

In this light, it is clear that it is absolutely impossible for clinicians to define the starting time of the acute infection based only on the presence of IgG and IgM. Moreover, the specificity and sensitivity of IgM are not 100%, but in the ranges of 93.3 to 100% and 77.5 to 99.1%, respectively, according to evaluations on the six ELISA kits most commonly used in the United States, in a study commissioned by the Food and Drugs Administration (FDA). 

The IgG avidity test is the laboratory resource currently accepted for the diagnosis of the approximate time when the initial infection occurred. This measures the affinity of IgG for binding to the antigens of *T. gondii*, and it tends to increase with the length of time that has elapsed since the initial infection [10–13]. A Mexican study on 100 pregnant women showed that there was no correlation between IgM levels and the IgG avidity test results. Thus, it was demonstrated in that study that the IgG avidity test is useful for inferring the infection phase and assisting in managing toxoplasmosis during pregnancy [14]. In this manner, avidity test results of up to 30% allow it to be said that the initial infection occurred not more than four months earlier. On the other hand, results of more than 60% indicate that the initial infection occurred at least six months earlier. Results of between 30 and 60% are inconclusive regarding the exact time of the initial infection. However, in practice, all the antibodies discussed above only allow conclusions consisting of suppositions and probabilities.

A prospective study assayed IgG avidity for toxoplasmosis among 146 pregnant women who presented with IgM positive for *T. gondii*. The patients underwent a multiplex nested polymerase chain reaction (PCR) for DNA of *T. gondii *in the amniotic fluid, maternal blood, and blood of the umbilical cord. Fifty (34.9%) of the pregnant women presented with low IgG avidity (less than 30%). The PCR performed on the amniotic fluid or performed at birth was positive in the cases of nine patients with low IgG avidity. Among these nine patients, only three presented with congenital toxoplasmosis. None of the pregnant women with high or threshold avidity presented positive PCR results in the amniotic fluid or congenital toxoplasmosis. There were no diagnoses of congenital toxoplasmosis among the patients with negative PCR results in the amniotic fluid. Thus, it was concluded from that study that using IgG avidity in association with PCR on the amniotic fluid was important for diagnosing congenital toxoplasmosis [15]. 

A prospective cohort study evaluated women with toxoplasmosis infection that had been detected in the prenatal screening at three centers, by means of real-time PCR on the amniotic fluid. PCR analysis was performed on 261 of the 377 patients who were included in the study. The sensitivity and negative predictive values were, respectively, 92.2% (95% confidence interval, CI: 81–98%) and 98.1% (95% CI: 95–99.5%). There was no significant association with the trimester of pregnancy in which the infection occurred. The specificity and positive predictive values were 100% in all three trimesters. Thus, real-time PCR was considered to be an important tool for predicting fetal infection due to *T. gondii* and for making more appropriate treatment decisions [16].

In a study conducted in France, at the University Hospital of Toulouse, 352 amniocentesis procedures were performed on pregnant women who were infected with *T. gondii*. The sensitivity and specificity of the diagnosis of congenital toxoplasmosis by means of PCR on the amniotic fluid were 91% and 99.5%, respectively. PCR was also performed on the placenta, which showed specificity of 52% and sensitivity of 99%. The specific IgG and IgM tests on the cord blood presented specificity of 91% and 92% and sensitivity of 53% and 64%, respectively. It was concluded in that study that PCR on the amniotic fluid has higher sensitivity and specificity for diagnosing congenital toxoplasmosis, thus enabling a more certain diagnosis of fetal infection [17]. A study conducted at the University of the Suez Canal, in Egypt, on 358 pregnant women with toxoplasmosis, showed sensitivity of 92.9% and specificity of 94.4% for PCR on the amniotic fluid, with a positive predictive value of 76.5% and a negative predictive value of 98.5% for the diagnosis of congenital toxoplasmosis [18].

Based on these studies, it has been accepted that the gold-standard examination for identifying fetal infection is PCR, which has high sensitivity and specificity when performed on amniotic fluid samples obtained from the sixteenth week of pregnancy onwards. In this manner, diagnosing the presence of DNA particles from *T. gondii* in this medium can be done with great certainty [19]. Since colonization of the fetal kidneys occurs around two to three weeks after invasion by the tachyzoites of *T. gondii *and production of the amniotic fluid occurs basically by means of fetal diuresis, it can be concluded that a positive PCR indicates the presence of infection in the fetus, which has been eliminating *T. gondii* DNA into the amniotic environment, in its urine. 

Attention has also been drawn to the possibility of reactivation of latent toxoplasmosis in immunosuppressed patients [20]. Maternal immunosuppression and contamination by *T. gondii* may also be related to higher risk of fetal disruption disorders [21]. Likewise, if it is considered that the pregnancy cycle naturally confers immunosuppression on pregnant women, it would not, in our view, be unreasonable to consider that reactivation of latent toxoplasmosis might occur in some pregnant women [22].

## 3. Treatment of Toxoplasmosis during Pregnancy

The World Health Organization and the Centers for Disease Control and Prevention recommend pyrimethamine, sulfadiazine, and folinic acid as the standard of care for people with congenital toxoplasmosis [23]. These medications were proven to be effective in a randomized prospective study called the National Collaborative Chicago Based Congenital Toxoplasmosis Study (NCCBTS). This study found that treatment with the three aforementioned medications significantly decreased adverse signs and symptoms associated with congenital toxoplasmosis, including ocular and central nervous system symptoms and sensorineural hearing loss [24]. For patients with sensitivity to sulfadiazine, clindamycin can be used in combination with pyrimethamine as an alternative [25].

When a diagnosis of acute maternal toxoplasmosis infection is made, spiramycin should immediately be administered to the mother, at a dose of 2 to 3 g per day. This medication does not go beyond the placental barrier, but it diminishes the risk of vertical transmission by up to 60%. Likewise, if PCR on the amniotic fluid is positive, institution of bacteriostatic therapy that crosses the placental barrier is essential, with the aim of diminishing the fetal malformations that result from acute toxoplasmosis. The following drugs can then be administered: sulfadiazine, at a dose of 3.0 g per day, and pyrimethamine, at a dose of 50 mg per day [26]. However, these medications have high teratogenic potential, since they noticeably diminish fetal serum folate synthesis. It is known that insufficient concentration of serum folates noticeably diminishes synthesis of the enzyme tetrahydrofolate reductase. This enzyme acts within the cell medium to transform homocysteine, which is a cytotoxic and teratogenic agent, into methionine, which is an essential amino acid. High tissue concentration of homocysteine is therefore one of the causes of higher incidence of structural abnormalities in fetuses [27]. For this reason, the administration of sulfadiazine and pyrimethamine should not be continuous and should be alternated with spiramycin administration every three weeks. In addition, a precursor of serum folate synthesis should be administered: folinic acid, at a dose of 10 to 20 mg, two to three times a week, while the patient is receiving sulfadiazina and pyrimethamine. Administration of these three drugs decreases the risk that the fetus might develop disruption abnormalities due to* T. gondii* infection, by 70% [28]. However, a major adverse effect of this treatment regimen is bone marrow suppression [29]. Bone marrow suppression leads to neutropenia, anemia, and thrombocytopenia. This adverse effect may be avoided with the simultaneous administration of folic acid during treatment [29, 30]. Despite these preventative measures, weekly monitoring of cell counts and platelet counts should be done to assess the level of marrow suppression and adjust these medications as necessary [30].

Recently, the Society of Obstetricians and Gynecologists of Canada proposed a clinical practice guideline to the toxoplasmosis in pregnancy. This guideline reinforces the conclusion that the combination of pyrimethamine, sulfadiazine, and folinic acid should be offered as treatment for women in whom fetal infection has been confirmed or is highly suspected, usually by a positive amniotic fluid PCR (level I-B of evidence). Women who are immunosuppressed or HIV-positive should be offered screening because of the risk of reactivation and toxoplasmosis encephalitis (level I-A of evidence) [31]. The necessity of the treatment of *T. gondii* in human immunodeficiency-virus infected pregnant women is proved by several case reports [32–34].

In study realized in Brazil that presents high incidence of toxoplasmosis in pregnancy, Porto and Duarte [35] analyzed the correlation between toxoplasmosis in pregnant women and incidence of congenital toxoplasmosis. Among the pregnant women classified as confirmed cases of toxoplasmosis in pregnancy (*n* = 19), the congenital toxoplasmosis risk was six times greater than that in the probable/possible group. No case of congenital toxoplasmosis was identified in the group of pregnant women classified as unlikely to have toxoplasmosis in pregnancy. The children with no prenatal treatment (46.2% *n* = 242/524) presented a risk almost of congenital toxoplasmosis three times greater than the treated children (odds ratio—OR 2.77; 95% CI 1.54–4.97; *P* = 0.001). Complete prenatal treatment was identified as a protecting factor for congenital toxoplasmosis (OR 0.35; 95% CI 0.19–0.65; *P* = 0.001) [35].

In study realized in Germany with 685 pregnant women and 33 children with positive serological tests for *T. gondii* infection, the overall transmission rate of *T. gondii* from the infected mother to her child was 4.8%. The prenatal treatment with spiramycin until sixteenth week was followed by at least 4 weeks of combination therapy with pyrimethamine, sulfadiazine, and folinic acid independent of the infection stage of the fetus [36]. These results are consistent with other studies in which only a small study population was included [37, 38]. 

Other alternative proposed treatment to toxoplasmosis in pregnancy is the spiramycin/cotrimoxazole association. This association was used in 76 pregnant women with positive serological tests for* T. gondii*. The treatment consisted of spiramycin 3,480 g 4 times a day, cotrimoxazole 960 mg (sulfamethoxazole 800 mg plus trhmetroprim 160 mg) twice a day, and folinic acid 4 mg/day. The intrauterine transmission rate was only 2.6% (two babies) and none of them showed signs or symptoms of congenital infection at birth or during followup. The treatment did not need to be stopped in any mother because of adverse drug effects [39]. In a case report, Tamaru et al. [40] used azithromycin as fetal therapy in a case of severe symptomatic fetal toxoplasmosis. The ultrasound realized at 23 weeks of pregnancy evidenced fetal ascites, cardiac effusion, cardiomegaly, enlarged lateral ventricles, and thickened placenta. Sulfadoxine (500 mg/day) and pyrimethamine (25 mg/day) were administered from 25 to 27 weeks. Cyclic administration of azithromycin (500 mg/day for 3 days followed by an interval of no medication for 4 days) was subsequently started at 28 weeks and continued for 3 weeks followed by an interval of no medication for a week until delivery. Then cyclic administration of acetylspiramycin (1.2 g/day for 3 weeks followed by an interval of no medication for 2 weeks) was combined with azithromycin for 31 weeks. Fetal ascites, cardiomegaly, enlarged lateral ventricles, and thickened placenta persisted until delivery; cardiac effusion of the fetus completely disappeared at 29 weeks, which the authors considered to be the partial effect of administration of azithromycin started at 28 weeks. 

**Figure 1:**
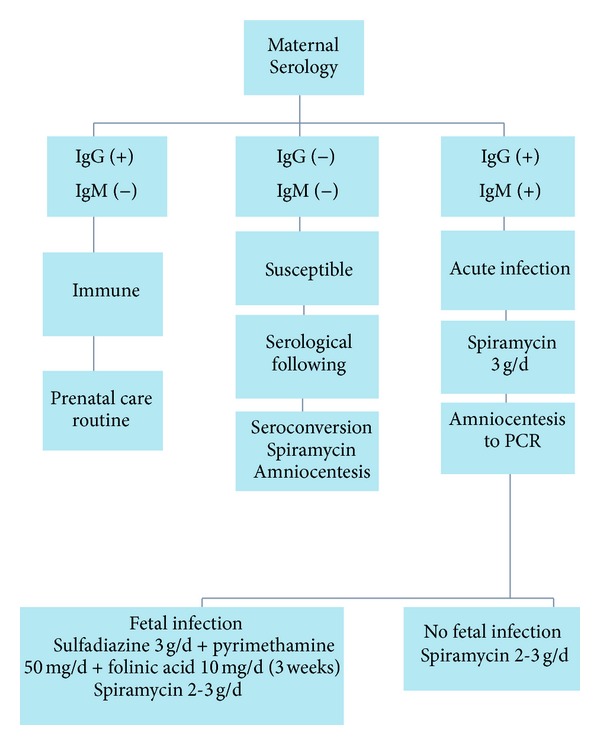
Diagram of serological screening of toxoplasmosis during pregnancy and its treatment.

Figure 1 shows a diagram with the sequence of serological screening of toxoplasmosis during pregnancy and its treatment. 

## 4. Conclusion

From a critical analysis on the data presented, we can conclude that it is essential to have results available from the IgG avidity test and from PCR on the amniotic fluid, so that treatment of pregnant women affected by *T. gondii* can be adequately managed. Without these test results, no conclusions regarding the presence or absence of vertical transmission of *T. gondii* to the fetus can be reached and doubts will remain regarding whether medications that cross the placental barrier, to treat the fetus, should be introduced. This uncertainty therefore could condemn a fetus that might have become contaminated across the placenta to develop a variety of congenital abnormalities, with serious postnatal repercussions for the family and for society as a whole, given that the ensuing costs to the public healthcare system are far from small.
